# Analysis model of college students' mental health based on online community topic mining and emotion analysis in novel coronavirus epidemic situation

**DOI:** 10.3389/fpubh.2022.1000313

**Published:** 2022-09-13

**Authors:** Zuqin Lu

**Affiliations:** ^1^Department of Special Education, School of Educational Sciences, Lingnan Normal University, Zhanjiang, China; ^2^Guangdong Provincial Key Laboratory of Development and Education for Special Needs Children, Lingnan Normal University, Zhanjiang, China

**Keywords:** COVID-19 epidemic situation, university student, psychoanalysis, online community, text data mining, sentiment analysis

## Abstract

Under the epidemic situation of COVID-19, university students have different levels of anxiety, depression, and other psychological problems, and these differing levels present different challenges. Therefore, universities and relevant departments should carry out accurate psychological health education for university students. Through research, this paper found that students' psychological problems during the COVID-19 epidemic were mainly reflected in four aspects: depression, interpersonal relationship, sleep and eating disorders, and compulsive behavior. Through the discussion of family of origin, self-awareness and motivation attribution, and social pressure, this paper analyzed the causes of psychological problems. The information resources of the network are usually unstructured data, and the text information, as the most typical unstructured data, occupies a large proportion. Moreover, this text information often contains users' emotional response to major events. In this paper, a data preprocessing system is designed, and three data preprocessing rules are defined: expression data conversion rules, data deduplication rules and invalid data cleaning rules. The characteristics of online community text data are analyzed, and the text feature extraction method is selected according to its characteristics. The results of this study show that the proportion of university students with psychological problems is about 23%, which is slightly higher than the research results during the non-epidemic period. This paper suggests that college students should master methods of self-regulation, improve their levels of physical exercise, improve their physical fitness, and establish and improve their defense mechanisms to alleviate psychological conflicts and pressures.

## Introduction

COVID-19, a new coronavirus, saw the outbreak of an acute infectious pneumonia, which was more infectious than previous iterations and had no particularly effective treatment. Because COVID-19 is highly contagious, most countries in the world have implemented strict travel bans and isolation measures. These measures have effectively controlled the spread of the epidemic, but at the same time, it inevitably caused collective anxiety and panic among the public. In the early stage of the epidemic, with the panic caused by public opinion, people over-focused on the development of the epidemic, realized the fragility of their lives, and felt anxious and pessimistic because they were worried about their health and regional security. With the benign control of the epidemic situation, the worry about health has gradually changed due to the notice of postponing the start of school in colleges and universities. Remote teaching has been affected by the speed of the network and the learning media, resulting in irritability. For example, in some areas, due to the limited network speed, some students can't synchronize their courses, and they can't get the teacher's answers in time when asking question, meaning they may be missing out on important learning. At the same time, they look at problems and themselves with a negative attitude, and lack motivation to do things. The epidemic situation in COVID-19 has had a great impact and influence on university students' psychological health. If the influencing factors are not explored, a series of negative events may be triggered.

Based on online community topic mining and sentiment analysis, this study explores the psychological status of university students and its influencing factors under the epidemic situation of COVID-19, and puts forward some suggestions in the post-epidemic period according to the analysis results. It is of great value to grasp the attitudes and opinions of netizens in the topic. Online hot topics are closely related to the real social sentiments, and the topics pushed by online communities in real time, as well as the infinite comments, reposts, and praises of online community users, make the speed of public opinion spread very quickly. Therefore, under the background of online community topics, online community users are prone to group polarization, and even lead to group events on the Internet or in real life (1–3). The comment emotion expressed by online users will not only affect the spread of public opinion on the whole topic, but also be influenced by other users. Words are the basic information for human beings to convey their feelings and express their thoughts, and they are also important external manifestations of the individual psychological state. Therefore, mining the individual's psychological state and emotional attitude when publishing content plays an important role in accurately identifying psychological health status (4). Text is a typical sequence data. If the context information of sentences can be captured, the emotional tendency of the text can be well-mined based on semantic understanding.

A person's state of mind plays a dominant role in their behavior, and can affect their learning and willingness and efficiency in learning activities (5). A healthy mental state is needed for learners to form rigorous logical thinking and carry out creative activities. University students are a special group, in the immature cognitive stage, prone to psychological problems such as anxiety and depression. Especially after the outbreak of the COVID-19 epidemic, many university students' psychological health problems have gradually become prominent due to many factors, such as reduced social activities, tight family relationships, high pressure of study and employment, etc. The extreme vicious incidents caused by university students' psychological problems are increasing gradually. How to accurately evaluate the psychological health status is not only an important task to ensure students' smooth learning activities, but also an important basis for intelligent psychological health education in colleges and universities (6, 7). When affected by external negative stimuli, individuals may experience depression, which lasts for a short time. Depressive tendency refers to an individual's depression caused by negative emotions or behaviors, which lasts for a relatively long time. This paper mainly evaluates the psychological tendency of university students, through processing and analyzing the multimodal network content data of students for a period of time, through which we can judge their psychological health state.

The innovation of this paper is as follows: firstly, this paper uses support vector machine and other algorithms to build a text classifier, and the classifier uses the captured data to train the emotion classification model. In this paper, the data preprocessing engine is used to de-duplicate, clean up, segment, and construct word vectors. The purpose of the data preprocessing engine is to format the original data into a training model in a desired form. The model designs a distributed online community crawler system. Sufficient data can be captured for the training model. The results show that the proportion of psychological problems among college students is about 23%, which is slightly higher than that in the non-epidemic period. This paper has made some suggestions to college students on mastering self-regulation methods, strengthening physical exercise, strengthening physique, establishing and improving defense mechanisms, and alleviating psychological conflicts and pressures.

The article is arranged as follows: The first section of this article introduces the related research of related scholars on online community emotional tendency analysis, the second section introduces the method of this article, the third section makes an experimental analysis of this method, and the fourth section is the full text summary.

## Related work

The content of this paper discusses emotional tendency analysis of online community texts. The purpose of emotional analysis is to discover people's emotional attitudes toward a topic event. Emotional attitudes can be divided into three categories: positive emotions, negative emotions, and neutral emotions. We can use natural language processing, text mining, computer linguistics, and other methods to identify and extract the subjective information in the original material, so as to analyze people's emotional attitudes.

Young et al. use some graph-based methods to distinguish emotional words in speech. They use the minimum cutting model and label iterative model method to complete emotional word recognition and text classification. Online community data contains not only text data, but also some expression data, and the expressions in online community texts are converted into text descriptions, so that users' emotional attitudes can be analyzed more accurately (8). Jabreel et al. put forward an affective computing framework, whose essence is to abstract the part-of-speech problems of affective words into graph optimization problems. They used the computer semantic dictionary, and based on this, they built a network graph of emotion words. Finally, the problem of emotion classification turned into the problem of dividing the generated undirected graph (9). Zhang et al. proposed an emotional semantic recognition method, which can be used to judge whether a certain word is an evaluation word, and then the classification method is used to analyze the positive and negative emotional polarity of these evaluation words (10). McGregor et al. believes that the essence of online public opinion is the basic attitude of the general public in cyberspace toward the rulers and holders of political values in the development and changes of dependent matters (11). Panwar et al. designed an emotional tendency analysis algorithm, which is an emotional polarity algorithm based on multiple features and phrase paths. At the same time, the algorithm is optimized by analyzing the publishing, forwarding, and commenting relationships of online community data (12). Moon divided vocabulary into two categories: general words and content words. Generally, general words are words that do not contain actual meanings, and the remaining words are defined as content words. In text analysis, we ignore the influence of common words, mainly consider the role of content words based on word frequency, and define the content words whose word frequency exceeds the threshold as effective words representing the theme of the article. For a sentence in a text, the greater the proportion of effective words in the whole sentence, the greater the significance of the sentence to the whole text (13). Junping et al., considering the vocabulary and structure of sentences, extracted nine semantic features that affect the sentiment of sentences, and used the method of manual and automatic acquisition to construct the ontology database of sentiment vocabulary, making a preliminary attempt on sentiment analysis (14). Fei et al., from the linguistic point of view, adopted the calculation method of giving priority to the definition of emotion tendency to get the semantic tendency of words in phrases, and analyzed the combination of words, and put forward the idea of a head word to calculate the tendency of words. This method laid the foundation for larger-grained text emotion analysis to identify the tendency of phrases that have a certain value (15). Faisal et al. put forward a text sentiment classification method based on weighted rough membership degree. This method uses the strength of feature tendency to define weighted rough membership degree, and applies it to a real car review corpus, and achieves good classification performance (16). Li et al. put forward an automatic recognition method of emotion evaluation unit based on syntactic path. This method automatically obtains the syntactic path to describe the modified relationship between the evaluation object and its evaluation words, and improves the system performance of emotion evaluation unit extraction by calculating the editing distance of the syntactic path. It has been applied in the field of electronic products and achieved good experimental results (17). Huang et al. proposed a text emotion classification method based on semantic understanding. By introducing emotional words into the recognition of emotional words, the concept emotion semantics was given, the emotional similarity of concepts was redefined, the emotional semantic values of words were obtained, and whether the emotional tendency of text was influenced by the appearance rules of semantic adverbs was analyzed. This method has improved the effective judgment of text emotional tendency (18).

## Design of online community sentiment analysis system

### Analysis and calculation of subjective and objective online community identification characteristics

**Figure 1:**
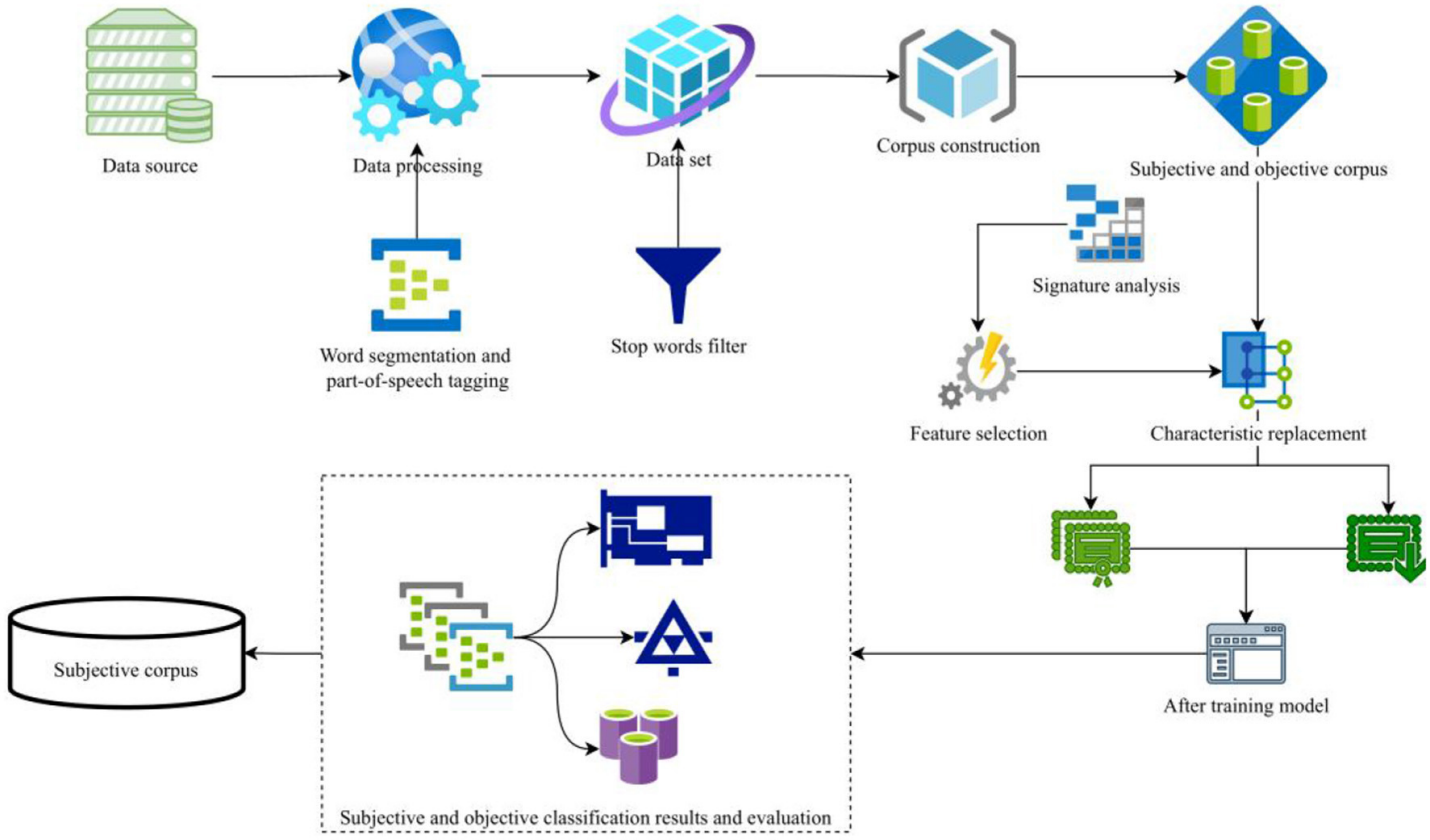
Subjective and objective online community identification scheme.

An emotion analysis system is a tool used to analyze the emotional tendency of text. Emotion can be divided into three categories: positive, negative, and neutral. Because of the large amount of Chinese corpus, it is impossible for the system to obtain all the text data at the same time, so the coverage of the training data in the system is very low, which requires the system to continuously update the training data. In English sentiment analysis system, word segmentation is not required for texts, but it is one of the necessary steps for Chinese text classification (19). The most important step in sentiment analysis is to choose the appropriate classification algorithm to construct a text classifier, which needs to be determined by combining the characteristics of data, algorithms, and text features. An online community features a kind of user-generated content, through which users can publish objective facts and their subjective attitudes. In the modern society with highly developed Internet, college students tend to publish text, images, expressions, and other modal data on social platforms to express their personal ideas and emotions. Different modal data have complementary effects and can provide more explanatory information. By integrating and understanding the multimodal data, a more comprehensive and systematic analysis and evaluation of students' mental health can be realized. Modal data refers to data containing two or more different forms or different sources. Therefore, this section regards the process of subjective and objective identification of online communities as the process of text classification, that is, the subjective online community and the objective online community are automatically classified by means of machine learning, and the results are evaluated and analyzed by the index of text classification performance evaluation. The process is shown in Figure 1.

Data acquisition is the basis of the experiment. It is very important to get the required online community data from the online community data, the authority of the data, and so on. In order to fully verify the research method proposed in this paper, the data set that has been publicly used by scholars or institutions at present is selected as the experimental data source in this experiment, thus avoiding the influence brought by individuals in the process of constructing corpus.

Useless information refers to the online community content that has nothing to do with the current topic, has no practical significance in spamming, or is websites, special symbols, etc. (20). Firstly, this paper analyzes the data set, understands the composition of the data set, and summarizes the characteristics of the useless information contained in it. Then, it designs relevant useless information filtering rules, builds a rule base, and finally filters out the useless information existing in the public data set. There are many words in an online community that are useless for subjective and objective online community identification. The existence of these words has no effect and will affect the efficiency of the system. Therefore, after word segmentation and part-of-speech tagging of online community texts, the online community is filtered by stop words with the help of the existing stoplist. The construction of a training set and test set is the foundation of a classification model. In this part, the experimental data are randomly divided into training set and testing set, and the online community in the training set and testing set is not repeated (21). According to the method of online community feature calculation, the space vector model is used to represent the features of each online community in the training set and the test set.

As the basic work of emotion analysis, subjective and objective online community identification is very important. How to analyze and select appropriate features to represent an online community is the key step of subjective and objective online community identification. This part analyzes the features of subjective and objective texts, and puts forward the calculation method of each feature. In this paper, the emotional polarity of 10,000 pieces of data is manually marked as the test corpus. Firstly, the emotional dictionary is used to classify the 10,000 pieces of data, and the results are shown in Table 1.

**Table 1:** Subjective and objective online community identification step.

**Actual**	**neg**	**neu**	**pos**
neg	1,893	662	530
pos	993	1,956	2,215
neu	3,479	2,607	878

Hot topics refer to topics that can break through the limitations of time, region, field, and audience, spread widely in the network, and can cause widespread public concern and hot discussion within a certain period of time, such as education, medical care, housing prices, employment, and other practical issues, as well as the behavior and moral problems of public figures, which can lead to the emergence of hot topics. Hot topics have far-reaching influence. More and more scholars try to find hot topics every time they look for new angles, but they ignore the complexity of the online community public opinion environment, and the influencing factors are relatively single. Compared with structured data in a database, text has limited structure or no structure at all. In addition, the content of the document is the natural language used by human beings, and it is difficult for a computer to process its semantics. Because of the particularity of text data source, the existing data mining technology cannot be directly applied to it, and the text needs to be analyzed. Metadata representing its features can be extracted, which can be saved in a structured form as an intermediate representation of the document. Its purpose is to scan and extract the required facts from the text. After extracting the features of the text information and storing them in the representation model, the text can be classified. The commonly used similarity calculation formulas are vector cosine angles as shown in Equations 1, 2.


(1) $$\begin{array}{l} dis\left(x_{i},y_{i}\right)=\sqrt{{\overset{N}{\underset{i=1}{\sum }}\left(x_{i}-y_{ij}\right)}^{2}} \end{array}$$

(2) $$\begin{array}{l} sim\left(x_{i},y_{i}\right)=\frac{\overset{N}{\underset{i=1}{\sum }}\left(x_{ij}\cdot y_{i}\right)}{\sqrt{\overset{N}{\underset{j=1}{\sum }}x_{ij}^{2}}\cdot \sqrt{\overset{N}{\underset{i=1}{\sum }}y_{i}}} \end{array}$$

After the isolated emotional words and dependency clusters extracted from each sentence are judged emotionally, the whole text can be classified emotionally. Considering that statements in different positions in the text may have different importance to the whole text, each statement should be given a weight, as shown in Equation 3.


(3) $$\begin{array}{l} weight\left(W_{i}\right)=\sum \frac{N+i+1}{N^{2}} \end{array}$$

The final sentiment classification results of the text will be comprehensively judged according to the isolated sentiment words extracted from each sentence, the sentiment discrimination results of dependency clusters, and the weight of each sentence. The scores of each emotion category of the text are calculated, and the text is judged as the emotion category corresponding to the highest score, as shown in Equation 4.


(4) $$\begin{array}{l} sorce\left(E_{i}\right)=\overset{N}{\underset{j=1}{\sum }}\left(weight\left(W_{i}\right)\cdot count\left(T_{i},W_{j}\right)\right) \end{array}$$

After normalization, in order to reduce the influence of the word frequency span on the computer, the formula is further optimized, as shown in Equation 5. Keyword density has an influence on SEO ranking optimization. If the keyword density has a great impact on the keyword ranking, then when we set the keyword density, the higher the density, the better the keyword ranking of the website. From the perspective of SEO optimization specialists, the ultimate goal of SEO optimization is to make the ranking of target keywords higher and better. There are some differences between the target keyword and other keywords in the keyword density setting.


(5) $$\begin{array}{l} W_{i,j}=\log\frac{\underset{k}{\sum }f_{j,k}}{f_{i,j}}\times \frac{N}{n_{i}} \end{array}$$

In this paper, the text is divided into sentences, and the emotional polarity of each sentence is further analyzed. The emotional polarity of a sentence can be calculated according to the polarity of emotional words in the sentence. Then, the emotional values of all sentences are divided and synthesized, so as to calculate the emotional polarity value of the text. The emotional value of online community texts can be determined by the emotional value of sentences, as shown in Equation 6.


(6) $$\begin{array}{l} F\left(s\right)=\sum F\left(s_{i,j}\right) \end{array}$$

All verbs and adjectives are used as emotional words, as shown in Equations 7, 8.


(7) $$\begin{array}{l} P_{i}=\frac{N_{p}}{\left(N_{f}+N_{p}\right)}*\frac{f_{pw}}{\left(f_{pw}+N_{wn}\right)} \end{array}$$

(8) $$\begin{array}{l} N_{wn}=\frac{N_{p}}{\left(N_{f}+N_{p}\right)}*\frac{fn_{pw}}{\left(fn_{pw}+Nn_{wn}\right)} \end{array}$$

In many cases, data are not linearly separable. In this case, this paper needs to map the data to a high-dimensional space, in which the originally non-linearly separable data become linearly separable (22). However, the dimensions of high-dimensional space will increase with the increase of data, and even infinite dimensions will appear in extreme cases, so it is difficult to directly calculate the model in high-dimensional space. Here, this paper uses kernel function to solve this problem. In order to make the emotional score more accurate, the emotional words with degree adverbs are added in this paper to calculate the emotional value, as shown in Equation 9.


(9) $$\begin{array}{l} {S_{w}}_{i}=\left(P_{si}-N_{si}\right)*\left(\partial +1\right)*N_{w} \end{array}$$

**Figure 2:**
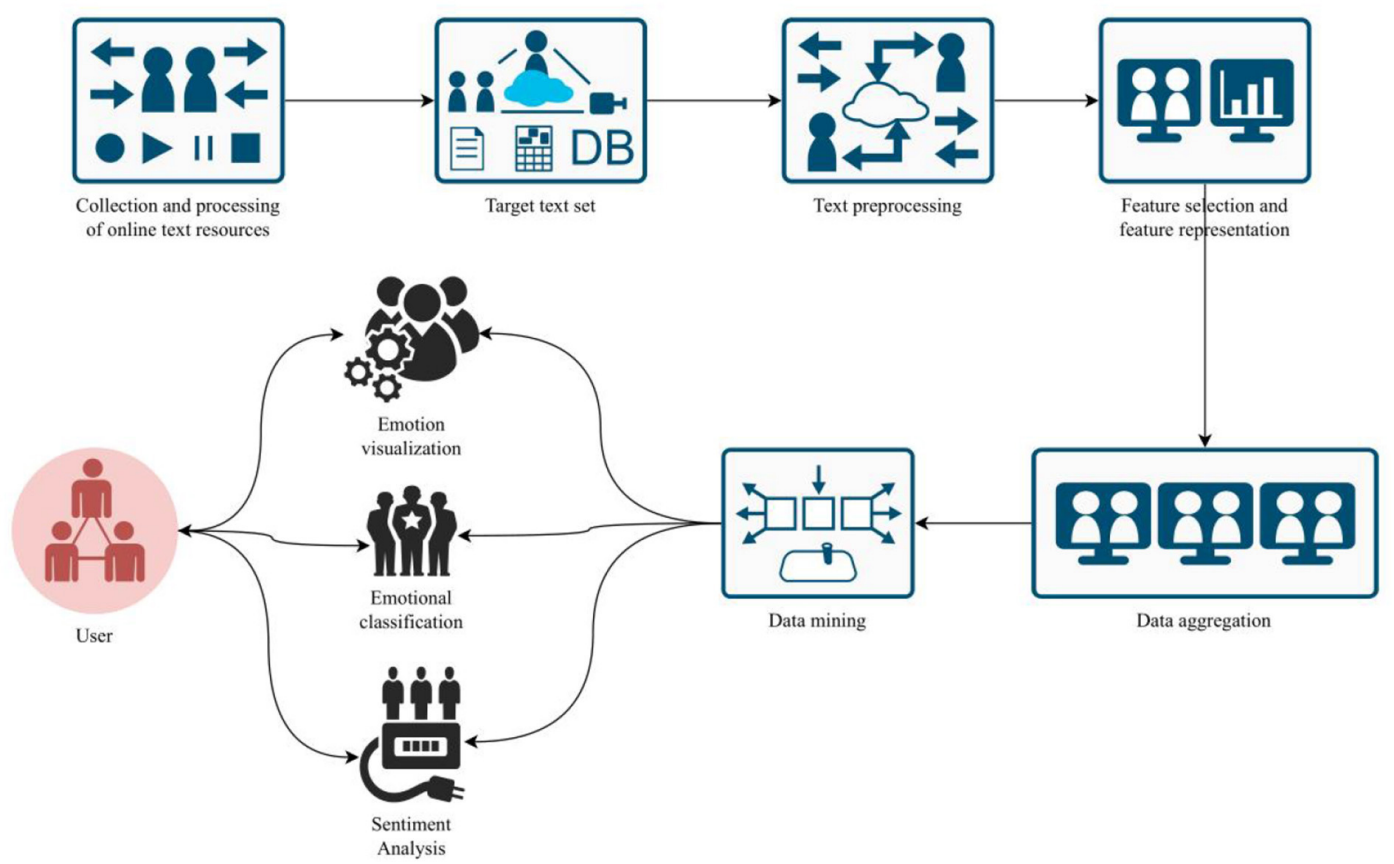
Basic processing of text mining.

Text mining is a process of processing semi-structured or unstructured natural language texts, and using certain techniques to discover and extract specific information from them. Web-based text mining firstly builds a target text set by collecting web-based text resources, and then processes the text set by using technologies such as text preprocessing, feature selection, feature representation, and data mining, and obtains the specific information that users need. The processing process is shown in Figure 2.

Natural language processing often converts words into one-hot coded word vectors, which are simple and easy to understand. However, when the order of magnitude of vocabulary is relatively high, it will lead to very high data sparseness and many dimensions of vectors, which will easily lead to dimension disaster, which is not conducive to the maintenance of computation (23). In this paper, the stoplist is used, and the online community text will be divided into words after Chinese word segmentation. Removing stop words means matching the words after word segmentation with the stoplist one by one. If they are the same, the word is designated as stop words, which should be deleted and filtered out. If the word does not appear in the stoplist, the word will be kept.

### Emotion analysis of online community based on text mining

In the environment of the COVID-19 epidemic, with the panic caused by public opinion, people were overly focused on the development of the epidemic, aware of the fragility of their lives, and worried about their own health and regional security, resulting in anxiety and pessimism. With the benign control of the epidemic situation, the worry about health has gradually changed due to the notice of postponing the start of school in colleges and universities. For example, in some areas, due to the limited network speed, some students can't synchronize their courses, and they can't get the teacher's answers in time when asking question, meaning they may be missing out on important learning. At the same time, they look at problems and themselves with a negative attitude, and lack motivation to do things. Affected by anxiety, some students have difficulty sleeping, and it is difficult to fall asleep at night. Some students can watch their mobile phones until 1 am or sleep for a shorter time than usual, and feel in a bad state during the day. At the same time, there is always a feeling of satiety when eating, and the appetite is obviously reduced. Interpersonal problems for students are mainly reflected in the fact that they have been with their families since the epidemic control, but there is less and less communication, and conflicts arise because of trivial matters. Current university students were born in the Internet age, and now they make friends, shop, get information, and kill time on the Internet as the most common medium, so the most common state is that they are constantly watching news or chatting on their mobile phones, lacking positive communication with their parents. Faced with this state, many parents usually reprimand. Students find it difficult to communicate with their parents, which leads them to think that their parents can't understand them.

**Figure 3:**
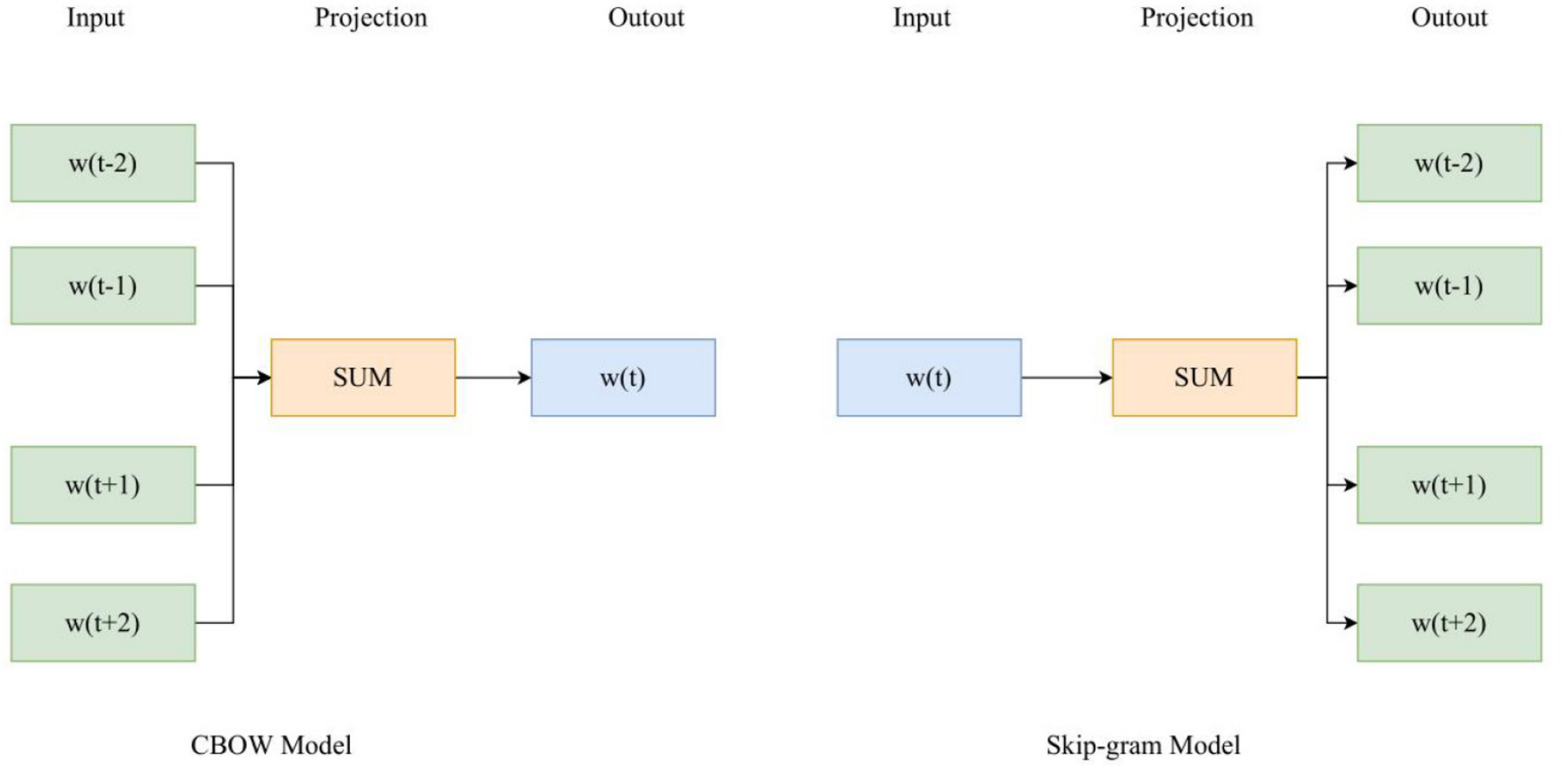
Model structure.

In the word embedding layer, the text is vectorized, and the text vectorization mainly processes the text data into a sequence vector form that can be directly received and processed by the BiLSTM layer. The model uses Word2Vec model to vectorize the text and generate word vectors. Word2Vec model has two kinds of CBOW and Skip-gram structures. CBOW structure is faster than Skip-gram structure, and Skip-gram structure is better than CBOW structure in expressing semantic information accurately, so this section uses Skip-gram structure, as shown in Figure 3.

Among them, CBOW model predicts the head word according to the words around the head word W(t). The first layer of CBOW model is the input layer, and the input value is the one-hot coding form of every word around it. The hidden layer only adds the weights of the output values, and there is no activation function for non-linear conversion. The dimensions of the output values are consistent with those of the input values. The third layer is the output layer. A binary tree corresponds to each word in the dictionary, and the conditional probability of a word is obtained through a Havermann tree. Affective analysis focuses on evaluative information with affective tendencies and has a wide range of applications. Emotion analysis is divided into three types according to different granularity: lexical emotion analysis, sentence emotion analysis, and document emotion analysis. This paper introduces sentence emotion analysis and its key issues. First, it briefly describes the task of sentence emotion analysis, then introduces the subjective and objective sentence classification methods and two subjective sentence emotion classification methods in sentence emotion analysis. The method is based on emotion words and machine learning method. Finally, three key issues in affective analysis, namely, the determination of lexical context polarity, the identification of evaluation topics, and the identification of opinion holders, are summarized. The emotional value of each sentence in the text after considering the sentence pattern features can be summarized according to different sentence patterns, as shown in formulas 10–13.


(10) $$\begin{array}{l} F^{\prime }\left(W_{i}\right)=F\left(W_{i}\right)\times \left(-\log3\right)+\left(-sim8\right) \end{array}$$

(11) $$\begin{array}{l} F^{\prime }\left(W_{t}\right)=F\left(W_{i}\right)\times \left(-\log15\right)+\left(-sim0.7\right) \end{array}$$

(12) $$\begin{array}{l} F^{\prime }\left(W_{j}\right)=F\left(W_{i}\right)\times \left(\log22\right) \end{array}$$

(13) $$\begin{array}{l} F^{\prime }\left(W_{k}\right)=F\left(W_{i}\right)+\left(-sim8.7\right) \end{array}$$

The emotional value of online texts is shown in Equation 14.


(14) $$\begin{array}{l} F^{\prime }\left(S\right)=\sum F\left(S_{i,j,k,t}\right) \end{array}$$

This paper studies the topic if online community sentiment analysis from three aspects: subjective and objective online community identification, online community sentiment tendency analysis, and online community sentiment analysis. This series of research is based on the construction of online community sentiment-analysis-related dictionaries. Therefore, this paper first builds online community sentiment-analysis-related dictionaries, and then, on the basis of in-depth analysis of online community corpus, researches subjective and objective online community identification, online community sentiment tendency analysis, and online community sentiment analysis are carried out. Before analyzing the emotional tendency and evolution of the collected online public opinion data, the collected data should be preliminarily processed, that is, the data should be de-duplicated, de-emptied, and de-advertised. First, the same content published by the same user an the online community at the same time should be deleted, so as to avoid the influence of repeated data on the effectiveness of subsequent sentiment analysis results; Then, the wrong online community data should be deleted, that is, the incomplete data or empty data. This paper makes necessary deletion of a large amount of junk information such as advertisements generated by the hot topic of an event. Finally, the online community data is repeatedly cleaned and checked, and the final results are shown in Table 2.

**Table 2:** Online community data collection fields.

**Collection field classification**	**Acquisition field**	**Use**	**Data type**
Weibo publishes user information	User name	Social network analysis	String
	User UID	Social network analysis	String
	User homepage	Evolutionary analysis	String
Weibo publishes content information	Release time	Evolutionary analysis	Date Tim
	Publishing mode	Other	String
	Publish content	Communication effect analysis	Double
	Publish URL	Communication effect analysis	Double
	Quantity of praise	Communication effect analysis	Double
	Forwarding quantity	Communication effect analysis	String
	Number of comments	Communication effect analysis	String

Text mining or document mining is a process of obtaining interesting or useful patterns from unstructured text information. Text mining covers a variety of technologies, including information extraction, information retrieval, natural language processing, and data mining. Its main purpose is to extract unknown knowledge from the original unused text. But text mining is also very difficult work, because it has to deal with fuzzy and unstructured text data. Therefore, it is a multidisciplinary field, covering information technology, text analysis, pattern recognition, statistics, data visualization, database technology, machine learning, and data mining. An emotion dictionary is the most common and basic technical means in the field of text emotion mining. Emotion classification methods based on an emotion dictionary are usually unsupervised learning methods, which mainly judge the emotion category of the whole sentence by obtaining the emotion tendency of each word in the sentence in an emotion dictionary. The most traditional classification method based on sentiment dictionary mainly depends on counting the number of positive sentiment words and negative sentiment words in sentences. The final sentiment classification result of the text will be comprehensively judged according to the sentiment discrimination result of isolated sentiment words extracted from each sentence, the dependency cluster, and the weight of each sentence. After filtering out high-frequency words with frequencies higher than 0.78 and low-frequency words with frequencies lower than 0.1, the number of topics should be divided into <10, an LDA topic model built, and the obtained consistency results should be averaged. The results are shown in Table 3.

**Table 3:** Average consistency of the number of different topics.

**Topic_num**	**2**	**3**	**4**	**5**	**6**	**7**	**8**	**9**	**10**
Coherence_avg	0.331	0.305	0.247	0.13	0.322	0.254	0.264	0.204	0.285

**Figure 4:**
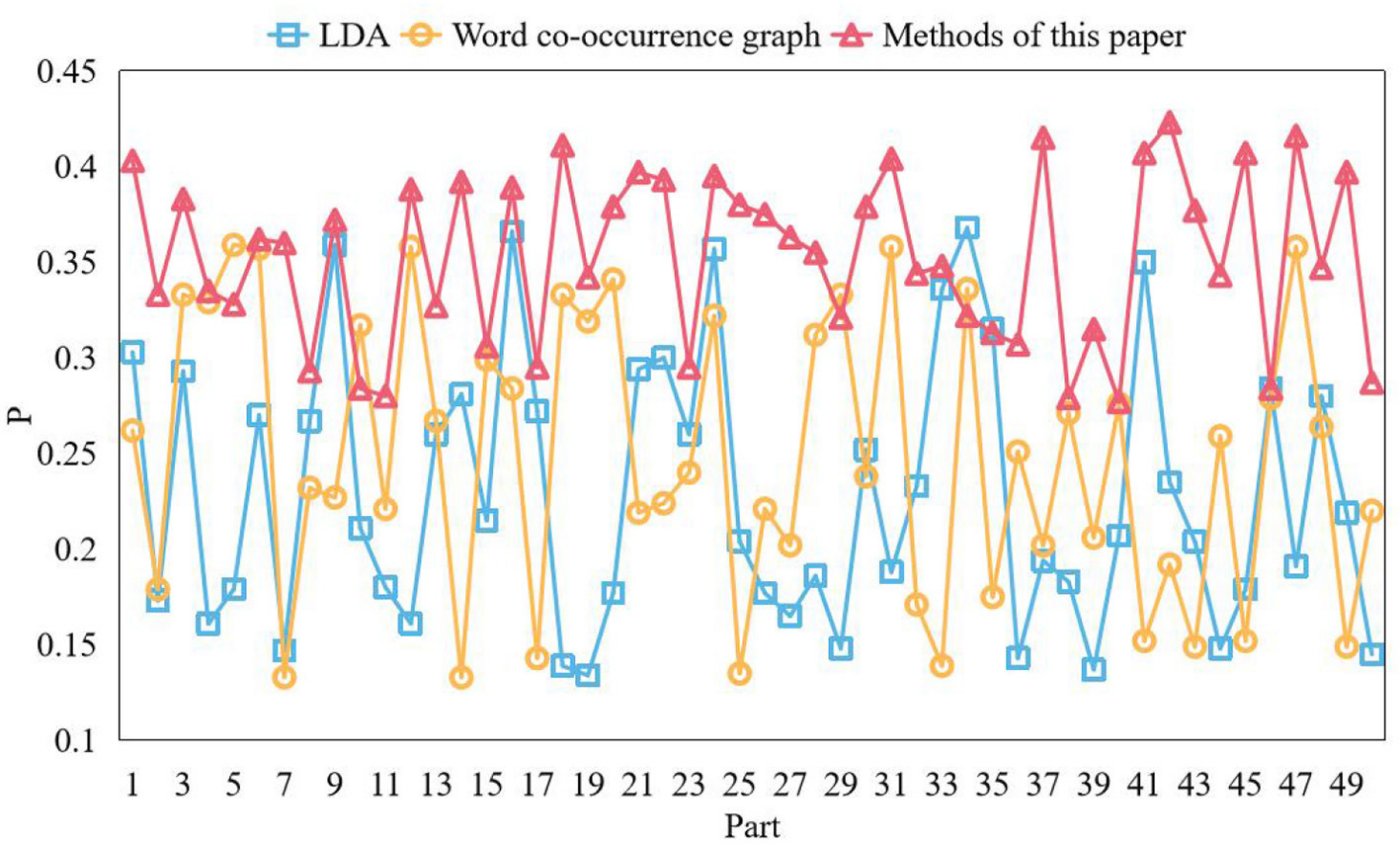
Accuracy comparison.

The extraction result is better when top ic_n um = 8. Therefore, in this section, the best_n = 8 is selected, and the keyword to p_n = 30 is selected for comparison and verification in each part of the corpus, that is, 236 keywords in each part. The keywords of the word co-occurrence graph should be determined. According to the word co-occurrence method, the first 500 high-frequency words are selected as the point set of the word co-occurrence graph. After calculating the co-occurrence degree of word pairs in this point set respectively, 236 keywords are also extracted from each part of the document after operations such as edge connection and cluster division. Then the key words of this method are determined. The accuracy of the results of the above three experiments are calculated, and the results are shown in Figure 4.

It can be seen from the figure that, however, the overall accuracy of the three models is not particularly high because of the weak correlation of the corpus, but this method has a better performance in extracting topic keywords, and can be used as a topic discovery algorithm. The first step in text mining is to obtain the corpus, and the second step is to preprocess the corpus. One of the important links of preprocessing is to segment the comment corpus, which aims to accurately split the Chinese character sequence into independent words, so that the words can be converted into vectors later. Compared with the English text, there are no separators between words. At present, the widely used word segmentation algorithms can be roughly divided into three word segmentation methods: understanding-based, string matching-based, and statistics-based. Syntactic analysis methods based on grammatical function matching generally mark the part of speech of words or phrases. Then, usually, a part of speech has many grammatical functions, and the grammatical functions of different words of the same part of speech may be very different. In some cases, the grammatical functions of words of different parts of speech may be the same. The Chinese syntactic analysis method based on grammatical function matching uses the grammatical function set of words and phrases to replace the existing part of speech markers and phrase markers.

**Figure 5:**
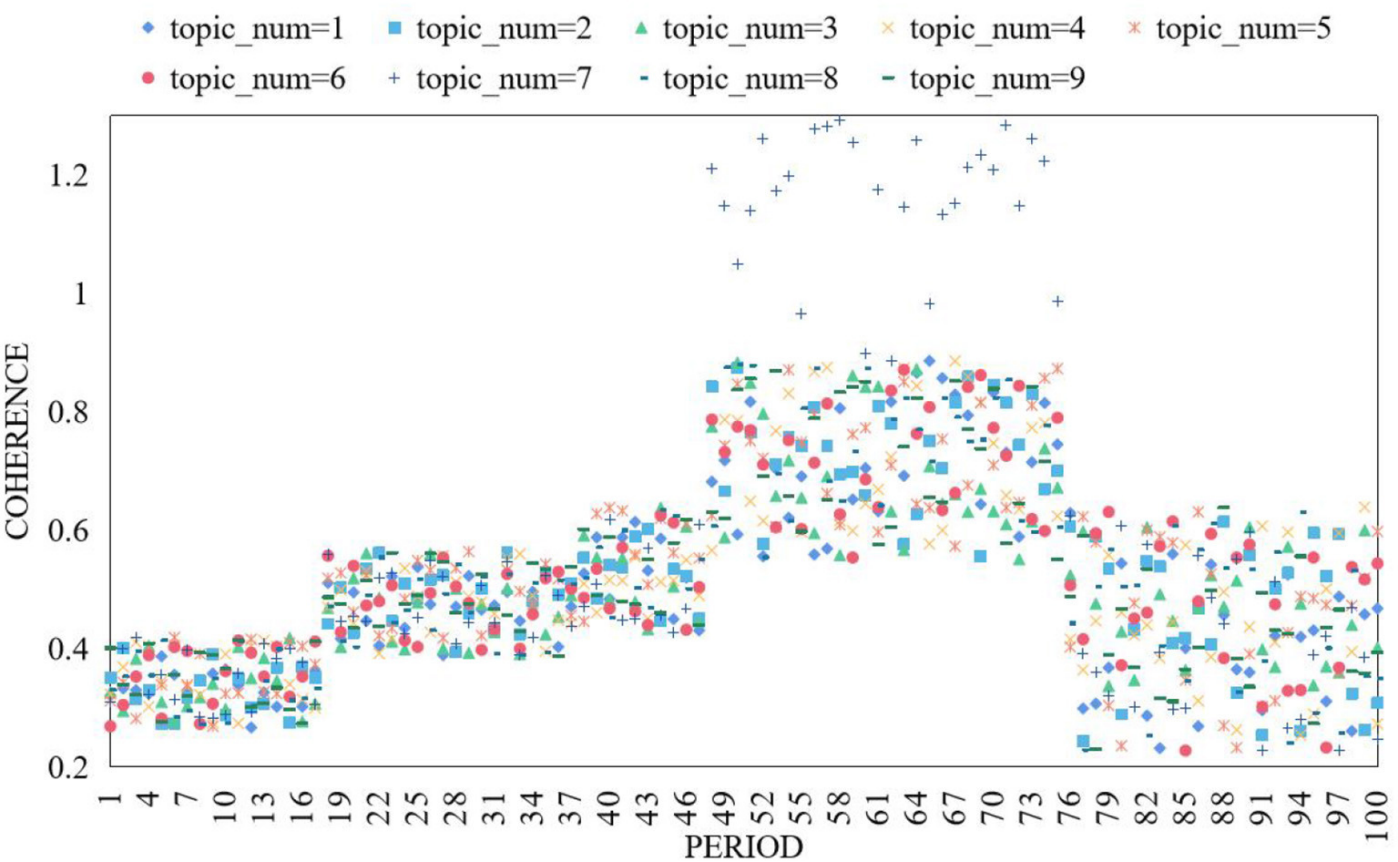
Consistency comparison of the number of different topics.

Comprehension-based word segmentation makes the computer do syntactic analysis and semantic analysis to simulate human's understanding of sentences, but because of the variability of language, this word segmentation method is not very advanced. The word segmentation method based on string matching needs a large enough matchable dictionary, which stores a large number of words, and then the computer will match the text sequence with these existing words, and one word segmentation will be completed. In order to determine the number of clusters of topic clustering, nine topics are modeled each week, and the consistent result broken line of each topic division per week is shown in Figure 5.

**Figure 6:**
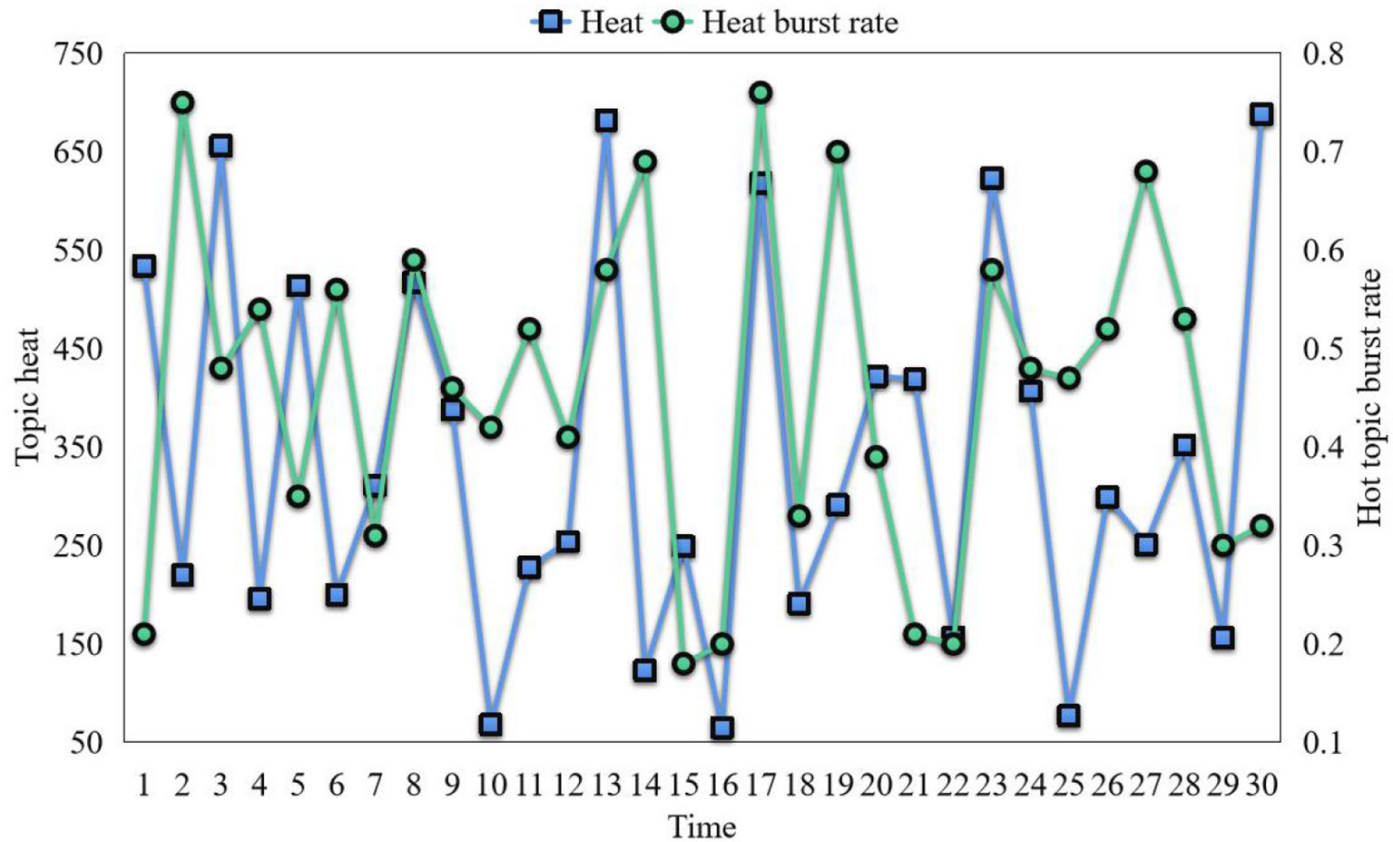
Topic heat and heat mutation rate.

It can be found from the figure that the fluctuation of the broken line with top i c_num = 7 is relatively small. The task of online community sentiment analysis is to classify online communities into three categories: positive, negative, and neutral. Because the content of online community is short and published randomly, the existing features can't represent the online community text well. Therefore, this paper attempts to analyze the online community corpus from the characteristics of Chinese online community corpus, and express the online community from three angles: emotional features, part-of-speech features, sentence features. Then, the SVM model with good classification effect is used to analyze the emotional tendency of online community, and different features are selected to compare the experimental results to verify the effectiveness of the features proposed by ontology. This study first analyzes the stylistic features of Chinese online communities, and takes them as an important basis for feature selection in subjective online community recognition experiments. Then, it selects emotional words, assertive verbs, modal particles, degree adverbs, and fixed part-of-speech structures. If the number of online communities of a topic does not exceed 100, it is considered that the topic's popularity is 0. After the calculation of popularity and mutation rate, the popularity of each topic in each period is ranked in descending order, and the mutation rate results are shown in Figure 6.

From the figure, we can see that the heat of the topic does exist. Mutation and the whole are hierarchical. If we only judge the hot topic according to the heat or mutation rate, there are indeed limitations. Then, when judging whether a topic is a hot topic, it should first be judged whether the heat of a topic is lower than 100; if so, the topic should be excluded, whether it is higher than 300; if so, it is a hot topic no matter what the mutation rate of the topic is; if the mutation rate quotient is 0.25, the topic should be excluded and all topics with lower heat than the topic; otherwise, it is a hot topic.

According to the corpus set of each keyword in different periods, the emotional words of each corpus should be found in the emotional dictionary, the PMI of the emotional words and the corresponding keywords calculated to judge the degree of correlation between the emotional words and the keywords, and the 10 emotional words with the highest degree of intimacy should be taken as the final emotional words of the keywords. The size of the dictionary directly determines the effect of word segmentation, and then a word segmentation method based on knowledge understanding is put forward. Word segmentation based on statistics is the most widely used method at present, and its main core is that the more times adjacent words appear at the same time, the greater the possibility of word formation. Therefore, the occurrence probability or frequency of adjacent words of a word can better reflect the credibility of the word. The combination frequency of adjacent words in the training text is calculated, and the mutual occurrence information between adjacent words should also be calculated. This mutual information reflects the closeness of the relationship between words. When the compactness exceeds a certain threshold, it can be considered that this word group constitutes a word. Because word segmentation is only the pre-work of the text sentiment analysis research in this paper, the commonly used word segmentation tool, stuttering word segmentation, is used for word segmentation.

## Emotion analysis experiment based on online community

Online community users can publish short text messages or multimedia content to their home pages anytime and anywhere through mobile phone short messages, web pages, instant messaging software, etc. At the same time, users can also use online communities to access information, which can be news reports, responses from others to their online communities, or online communities of people they care about. Through emotional analysis of an online community, users can not only know the social impact of the topic they care about, but also know the public's emotion and emotional state of the topic.

**Figure 7:**
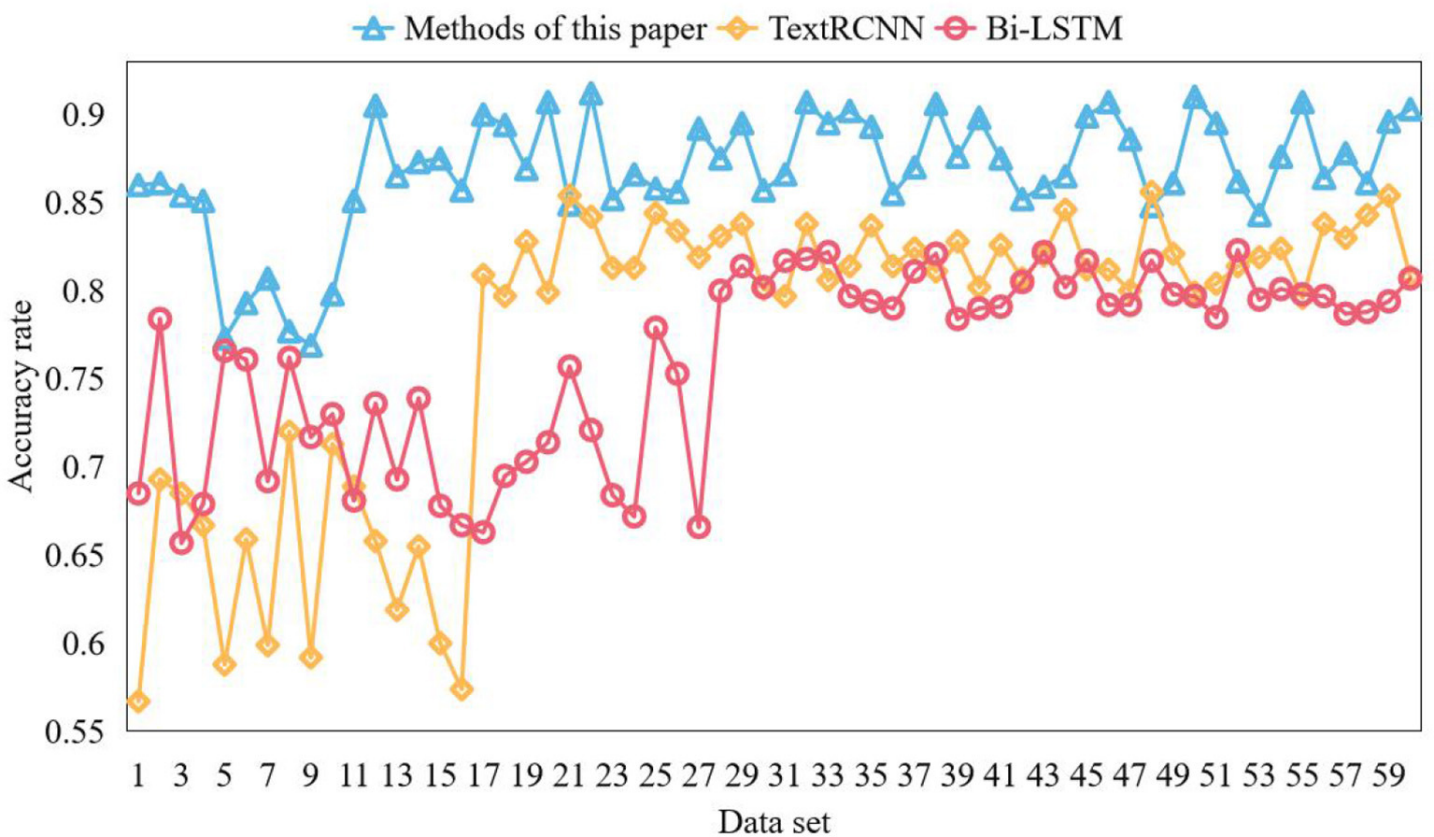
Comparison of model accuracy.

**Figure 8:**
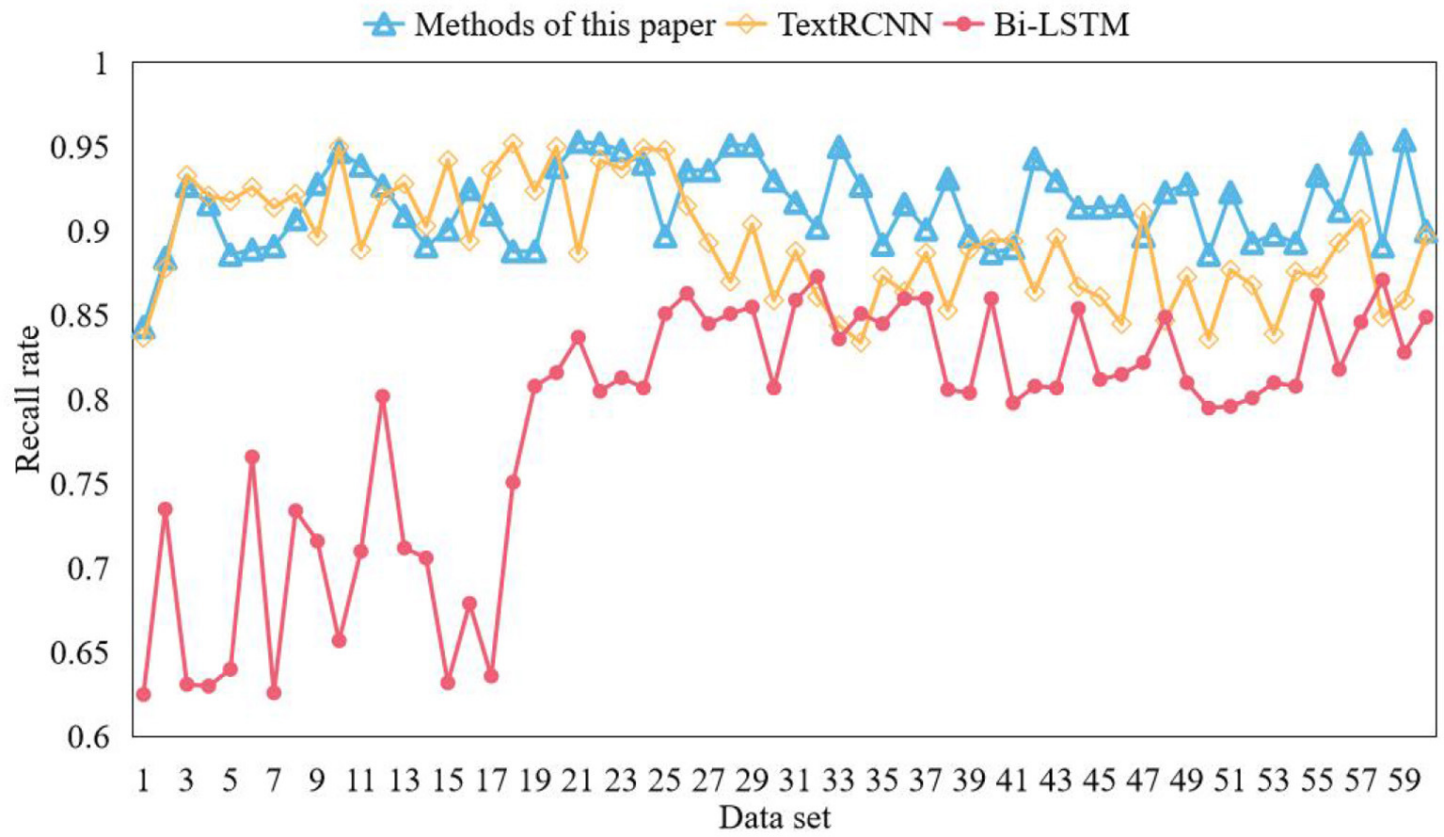
Comparison of recall rates of models.

This paper classifies online community text comments with emotion. In order to verify the effectiveness of the model, this paper uses the online community comment data set weibo_senti_100k to test the model. BiLSTM model and TextRCNN model are used for comparative experiments. Bilstm is a combination of forward LSTM and backward LSTM. LSTM model is composed of input gate, forgetting gate, cell state, and output gate added on the basis of RNN. During network training, information can be added or removed through the gate structure. Different neural networks can decide which relevant information to remember or forget through the gate structure on the cell state. In the textcnn network, the network structure is in the form of convolution layer + pooling layer, and the convolution layer is used to extract the features of n-gram type. For the models participating in the comparative experiment, the word vector model is used to vectorize the text, and the obtained word vector is input into the model for deep feature extraction. Finally, softmax classifier is used to classify the text. The emotional words of each corpus should be found in the emotional dictionary from the corpus set where each keyword is located, the PMI of the emotional words and the corresponding keywords calculated to judge the degree of correlation between the emotional words and the keywords, and the 10 emotional words with the highest degree of correlation taken as the final emotional words of the keywords. All PMI values are normalized to calculate the contribution of emotional words to keywords. Three contrast models are trained on the training set, and the training results are shown in Figures 7, 8.

In this paper, the online community texts are word-oriented quantized by Word2Vec model, and then the extracted feature vectors are used to obtain the context of the texts by BiLSTM model. At the same time, the Attention mechanism is introduced to express the importance of different features, and the model is further optimized. Finally, it is classified by softmax function. This model is used to classify online community comment texts, and the experimental results verify the feasibility and effectiveness of this model. Word vector only memorizes a relatively small amount of word information, while more semantic and syntactic information is memorized by hidden layer. Therefore, the dimension of word embedding need not be consistent with the dimension of hidden layer, and the parameter quantity can be reduced by reducing the dimension of word embedding. ALBERT adopted the factorization method to reduce the parameters. Suppose the size of the vocabulary is V, the embedded dimension of words is E, and the hidden dimension is H. Firstly, the one-hot vector is mapped to a low-dimensional space. ALBERT model achieves the purpose of reducing a large number of parameters by sharing all the parameters in the encoder with a slight loss of performance. This method can greatly improve the prediction accuracy without significantly increasing the computational complexity, and is insensitive to multicollinearity. The influence of each variable on the observed value of each node in the classification tree is calculated by Gini coefficient, and then the variables are sorted by this method model. According to the ranking results of variables' importance, the model of this method is fitted step by step, and the independent variable set with the smallest out-of-pocket estimation error rate is incorporated into the logistic regression model. The random seed number of the method model in this paper is set to 874, and the analysis results show that the total number of items and the order of importance of each variable in this method model from high to low are shown in Table 4.

**Table 4:** Multi-factor regression analysis of psychological status score.

	**Group**	**OR (95% CI)**
Grade	Freshmen and sophomores	2.726
	Junior and senior	3.861
Father's educational level	High school or technical secondary school	2.160
	University or above	1.379
Mother's educational level	High school or technical secondary school	1.967
	University or above	2.472
Have there been any major life events	No	0.601
	Yes	1.765

**Figure 9:**
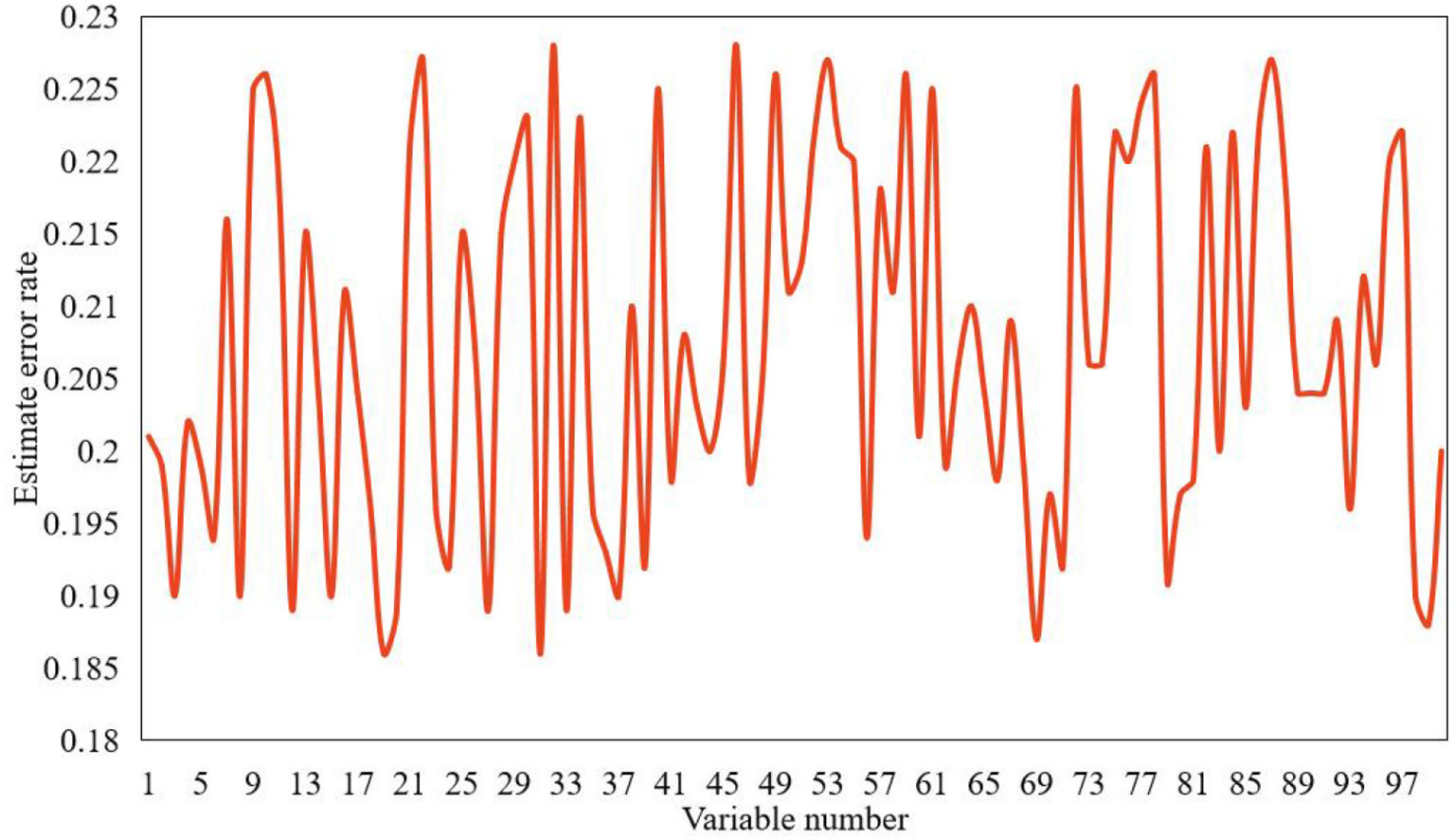
Error analysis of psychological status score.

According to the ranking results of independent variables' importance, the variable with the highest score will be predicted step by step, and the result of the method model analysis in this paper is shown in Figure 9.

In this paper, the emotional words extracted from the online community texts during the outbreak period are classified, the frequency of emotional words contained in the second-level emotional types during the outbreak period is obtained, and the proportion of the number of hot emotional phrases to the total number of newly arrived emotional phrases is finally obtained. On the online community platform, most of the data are text data, mainly including online community texts and online community comments. Compared with traditional data, Internet data has some remarkable characteristics, including huge data volume, diverse data types, and low data value density. These online community texts are people's opinions on some events and topics. People exchange views and attitudes through online communities. The traditional text emotion analysis method consumes a lot of human resources. However, the coverage of manually extracted features is limited and the artificial irrational behavior will affect the correctness of the results. Therefore, the traditional method is not universal. Deep learning has advantages such as automatic feature extraction, learning and correcting output, and processing nonlinear and complex data. The method of text emotion analysis is attracting the attention of many scholars in natural language processing. It can be predicted that the method of text emotion analysis will become an important trend in the research of text emotion analysis.

The results of this study show that the proportion of university students with psychological problems is about 23%, which is slightly higher than the research results during the non-epidemic period. Interacting through online communities has changed the traditional way of people's communication and interaction and daily living and learning habits. This way saves time and makes it easier and faster for people to get information. In the text of online community and related comments, most of the data are people's opinions and attitudes on some topics, which are valuable for analysis. For any topic, we can predict people's views and emotional attitudes toward events or topics by analyzing users' online communities and comments, and also predict the impact of events on society. Text sentiment analysis belongs to the category of information retrieval and natural language processing. By grasping the text data and analyzing the emotional tendency in the text, we can mine and predict people's emotional attitude toward a social event or topic, provide reference for the analysis and guidance of university students' psychological health, and predict the future development trend of things according to the analyzed emotional tendency.

## Conclusion

According to the characteristics of College Students' social network platform data, this paper constructs an automatic evaluation model of College Students' mental health based on multi-modal fusion calculation. The experimental results on the data set show that the accuracy of the multi-modal data model is significantly improved compared with the single-modal data. The results of this study show that the proportion of psychological problems among college students is about 23%, which is slightly higher than that in the non-epidemic period. Therefore, in the process of normalization of the epidemic situation, more attention should be paid to the psychological health of college students, and real-time monitoring of the psychological health of college students should be carried out. The pertinence and scientificity of mental health education should be improved, mental health problems discovered and prevented as soon as possible, problems effectively treated, and the mental health level of college students improved. The method proposed in this paper can effectively identify the psychological keywords of epidemic news. The Theme Evolution Law of the epidemic situation can be excavated, and form a cooccurrence knowledge map centering on “epidemic situation” and “prevention and control” and other keywords. At the same time, the emotion of the epidemic was positive. The nine time periods involved hot topics such as the epidemic, prevention and control, hospital, work, and service. The method of this paper can effectively explore the psychological health of college students, and summarize the evolution law and co-occurrence knowledge of College Students' psychological hot news. This paper can provide ideas for future disaster response and emergency and public opinion analysis.

## Data availability statement

The original contributions presented in the study are included in the article/supplementary material, further inquiries can be directed to the corresponding author.

## Author contributions

The author confirms being the sole contributor of this work and has approved it for publication.

## Funding

This work was supported by the phased achievement of the 13th Five-Year Plan of philosophy and social sciences in Guangdong Province in 2020: Research on the Relationship Between Family Support, School Support, and School Adaptation of Regular Primary School Students (No.: GD20XJY27) and the phased achievement of Guangdong Provincial Key Laboratory of Development and Education for Special Needs Children in 2021: Research on the Pre-Braille Curriculum for Preschool Children with Visual Impairments (No.: TJ202102).

## Conflict of interest

The author declares that the research was conducted in the absence of any commercial or financial relationships that could be construed as a potential conflict of interest.

## Publisher's note

All claims expressed in this article are solely those of the authors and do not necessarily represent those of their affiliated organizations, or those of the publisher, the editors and the reviewers. Any product that may be evaluated in this article, or claim that may be made by its manufacturer, is not guaranteed or endorsed by the publisher.
